# Latitudinal patterns and their climate drivers of the *δ* ^13^C, *δ* ^15^N, *δ* ^34^S isotope signatures of *Spartina alterniflora* across plant life-death status: a global analysis

**DOI:** 10.3389/fpls.2024.1384914

**Published:** 2024-05-31

**Authors:** Dongjie Zhang, Hui Wang, Xuepeng Liu, Kang Ao, Wenjun He, Tongxin Wang, Mingye Zhang, Shouzheng Tong

**Affiliations:** ^1^ Shandong Key Laboratory of Eco-Environmental Science for the Yellow River Delta, Shandong University of Aeronautics, Binzhou, Shandong, China; ^2^ School of Geographical Sciences, Northeast Normal University, Changchun, Jilin, China; ^3^ Northeast Institute of Geography and Agroecology, Chinese Academy of Sciences, Changchun, Jilin, China

**Keywords:** latitudinal pattern, biological invasion, isotope signature, coupling relationship, climate drivers

## Abstract

Isotopic signatures offer new methods, approaches, and perspectives for exploring the ecological adaptability and functions of plants. We examined pattern differences in the isotopic signatures (*δ* ^13^C, *δ* ^15^N, *δ* ^34^S) of *Spartina alterniflora* across varying plant life-death status along geographic clines. We extracted 539 sets of isotopic data from 57 publications covering 267 sites across a latitude range of over 23.8° along coastal wetlands. Responses of isotopic signatures to climate drivers (MAT and MAP) and the internal relationships between isotopic signatures were also detected. Results showed that the *δ* ^13^C, *δ* ^15^N, and *δ* ^34^S of *S. alterniflora* were -13.52 ± 0.83‰, 6.16 ± 0.14‰, and 4.01 ± 6.96‰, with a range of -17.44‰ to -11.00‰, -2.40‰ to 15.30‰, and -9.60‰ to 15.80‰, respectively. The latitudinal patterns of *δ* ^13^C, *δ* ^15^N, and *δ* ^34^S in *S. alterniflora* were shaped as a convex curve, a concave curve, and an increasing straight line, respectively. A decreasing straight line for *δ* ^13^C within the ranges of MAT was identified under plant life status. Plant life-death status shaped two nearly parallel decreasing straight lines for *δ* ^34^S in response to MAT, resulting in a concave curve of *δ* ^34^S for live *S. alterniflora* in response to MAP. The *δ* ^15^N of *S. alterniflora* significantly decreased with increasing *δ* ^13^C of *S. alterniflora*, except for plant death status. The *δ* ^13^C, *δ* ^15^N, and *δ* ^34^S of *S*. *alterniflora* are consistent with plant height, stem diameter, leaf traits, etc, showing general latitudinal patterns closely related to MAT. Plant life-death status altered the *δ* ^15^N (live: 6.55 ± 2.23‰; dead: -2.76 ± 2.72‰), latitudinal patterns of *S*. *alterniflora* and their responses to MAT, demonstrating strong ecological plasticity and adaptability across the geographic clines. The findings help in understanding the responses of latitudinal patterns of the *δ* ^13^C, *δ* ^15^N, and *δ* ^34^S isotope signatures of *S. alterniflora* in response plant life-death status, and provide evidence of robust ecological plasticity and adaptability across geographic clines.

## 1 Introduction

Biological invasion is a global problem that poses a threat to local plant communities, alters the patterns of macrobenthic animals, affects the habitat and food sources of migratory birds, and has negative effects on material circulation, energy flow, socio-economic activities, and other aspects of coastal wetland ecosystems (Sampaio et al., 2021; Zhang et al., 2021a; Li et al., 2022; He et al., 2023). Most invasive species can survive in large geographic areas where they germinate, colonize, grow, reproduce, expand, and develop corresponding adaptation strategies across latitudes (Liu et al., 2017 and 2022; Chen et al., 2023). Latitudinal gradients in abiotic factors, including temperature, precipitation, and soil physicochemical properties, increase environmental heterogeneity, shape the plant traits of invasive species, and form latitudinal patterns of plant communities coexisting with local and invasive species (Kirwan et al., 2009; Liu et al., 2020; Cheng et al., 2022). Altered plant traits (including plant height, stem diameter, leaf traits, specific leaf area, dry matter content, shoot density, productivity, reproductive traits, ecostoichiometry, etc.) through phenotypic plasticity promote the probability of successful invasion by invasive species, recognized as important mechanisms for invasion (Maron et al., 2004; Kirwan et al., 2009; Liu et al., 2017; Liu et al., 2020; Liu et al., 2020; Zhang et al., 2021a; Chen et al., 2022; Cheng et al., 2022; Liu et al., 2022; Zheng et al., 2022). In recent decades, research on plant traits has made good progress, and the introduction of new technologies, represented by stable isotopes, has provided new methods and ideas for exploring the mechanisms of invasive species (Hill et al., 2018; Watson et al., 2018; Wang et al., 2023). Latitudinal patterns of isotope signatures in invasive plants across coastal wetlands have become a new topic.

Stable isotopes, a type of natural isotopes existing in organic organisms, are non-radioactive and stable (Lin and da SL Sternberg, 1993; Feng et al., 2018; Chen et al., 2023). They typically possess a relatively long half-life and are not limited by their duration. Stable isotopes offer numerous advantages, including easy operation, high sensitivity, safety, non-toxicity, rapid detection, accurate results, and relative stability (Spivak and Reeve, 2015). They primarily exploit the same physiological and biochemical properties of labeled compounds and their corresponding non-labeled compounds to trace the intricate and variable chemical reactions and biological processes in organic organisms. This facilitates the observation of metabolic patterns and bioavailability of the tracked substance in the organism by monitoring changes in isotopic ratios (Bai et al., 2012; Kou et al., 2020; Xia et al., 2023a). Carbon (C), nitrogen (N), and sulfur (S) are essential components in plant tissues, and their corresponding stable isotopes are closely associated with plant physiological metabolism, growth, and development processes (Hill et al., 2018; Liu et al., 2021; Wittyngham et al., 2023; Xiong et al., 2023). Plant photosynthesis plays a crucial role in the fractionation effect of *δ* ^13^C. The *δ* ^13^C can be utilized to study the chemical development process of biogeography, the allocation of photosynthetic carbon in plants, the identification of plant photosynthetic pathways, and the evaluation of water use efficiency and biomass changes in plants characterizing the litter decomposition process (Liu et al., 2018; Zhang et al., 2021a; Xia et al., 2023b). The *δ* ^15^N isotope is commonly employed to assess the utilization efficiency, loss, nutrient uptake, and transport process of nitrogen elements in plant organisms and even plant communities (Hill et al., 2018; Xia et al., 2023a). The *δ* ^34^S isotope in plants can provide crucial information on the absorption of atmospheric sulfides by plants and the metabolism of sulfur in plants, offering a powerful tool for a deeper understanding of the interaction between organisms and their living environment (Guo et al., 2020; Jinks et al., 2020; Guiry et al., 2022). Stable isotopes, especially their local and global patterns, play a significant indicative role in monitoring short-term and long-term environmental changes in the biosphere. Applying stable isotopes in the study of invasive plant species in coastal wetlands and understanding the information reflected by isotopic changes contribute greatly to revealing the invasion mechanism.

*Spartina alterniflora*, recognized as a typical invasive plant, is a perennial monocotyledonous plant belonging to the Poaceae family (Cheng et al., 2022; Jia et al., 2022). It possesses extensive roots and robust reproductive capabilities, commonly growing in the intertidal zones of estuaries, bays, coastal mudflats, and tidal-influenced beaches worldwide (Humphreys et al., 2021; Mao et al., 2023). *S. alterniflora* plays a significant role in ecological and economic benefits, including carbon and nitrogen fixation, wind and wave prevention, embankment and beach protection, soil improvement, and the expansion of animal and plant habitats (Lu et al., 2020; Meng et al., 2020). However, the negative ecological impact of *S. alterniflora* invasion is becoming increasingly severe, affecting the structure and composition of biological communities. This invasion damages the composition and transmission of the food chain in coastal wetland ecosystems, leading to extreme instability in the ecological environment of coastal wetlands (Jinks et al., 2020; Li et al., 2022; Jia et al., 2022). Due to its high tolerance to salinity, rapid growth rate, and extensive range, *S. alterniflora* alters surrounding environmental factors by secreting a significant amount of salt into the environment during high-intensity transpiration. Simultaneously, it is an invasive species well-adapted to the coastal wetland environment, suppressing the growth of local plants in the surrounding environment by seizing living space and resources, thereby changing the structure and function of wetlands (Liu et al., 2017; Mao et al., 2019; Liu et al., 2022; Liu et al., 2020; Humphreys et al., 2021). Previous studies on *S. alterniflora* have focused on the invasion mechanism, physiological responses under different driving forces, competition mechanisms between native and invasive species, ecological prevention and control measures, and comprehensive analysis and utilization of biomass energy (Courtney et al., 2016; Ma et al., 2019; Hessini et al., 2022; Li et al., 2022). Some studies have explored *S. alterniflora* stable isotopes, systematically analyzing element dynamics during the decomposition process of *S. alterniflora* residues and changes in plant-soil element pools and nutrient transport processes. He et al. (2023) compared the trophic contribution of *S. alterniflora* to the macrozoobenthos between the dense *S. alterniflora* area and adjacent tidal bare mudflat in the Hepu coast by analyzing *δ* ^13^C and *δ* ^15^N. Wang et al. (2024) found that *S. alterniflora* invasion increased the values of *δ* ^13^C and *δ* ^15^N, as well as organic matter decomposition. Wu et al. (2024) used a ^15^N stable isotope dilution technique to investigate sediment gross N mineralization and NH_4_^+^ immobilization under aerobic and anaerobic conditions in *S. alternifora* communities. However, there has been little attention to the distribution pattern and influencing factors of *δ* ^13^C, *δ* ^15^N, and *δ* ^34^S in *S. alterniflora* across latitudes (Kinney and Valiela, 2018; Chen et al., 2021; Zhang et al., 2021 and Zhang et al., 2021). Additionally, there is minimal research on the impact of the life-death status of *S. alterniflora* on isotopic distribution.

Here, we compared the latitudinal patterns of the isotope signatures (*δ* ^13^C, *δ* ^15^N, *δ* ^34^S) of *S*. *alterniflora* and their responses to climate drivers under different plant life-death status by collecting isotopic data from literature records at 267 sites across coastal wetlands. Building upon previous findings on plant traits of *S. alterniflora*, we tried to address the following questions: (1) Do the latitudinal patterns of the isotope signatures of *S. alterniflora* vary with life-death status? (2) How is the geographical variation in *S. alterniflora* isotope signature influenced by mean annual temperature (MAT) and mean annual precipitation (MAP)? (3) Have strong coupling relationships formed between the isotope signatures of *S. alterniflora*? We hypothesized that: (1) The latitudinal patterns of the *S. alterniflora* isotope signature under the life status would outperform the dead status; (2) Geographical variation in *S. alterniflora* isotope signature would be driven by MAT and MAP; (3) The *δ* ^15^N and *δ* ^34^S of *S. alterniflora* would respond to the corresponding *δ* ^13^C in a linear or nonlinear form.

## 2 Materials and methods

### 2.1 Literature sources and screening

**Figure 1:**
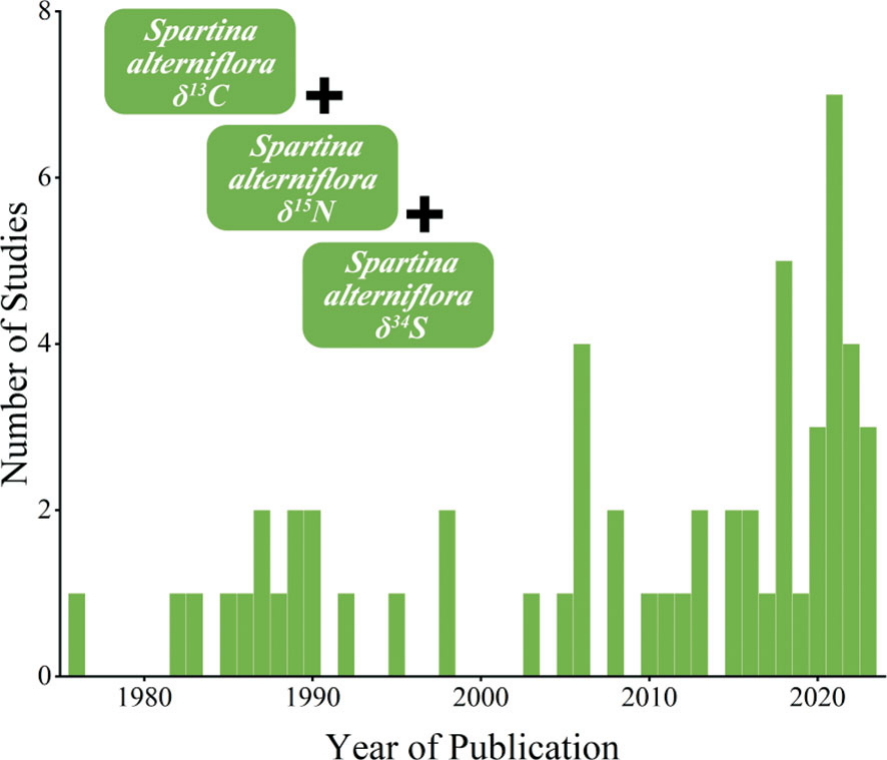
Number of relevant studies included in this study (*N*=57) published per year (1978–2023). The figure style reference Mason et al, 2023.

We conducted a systematic literature search for peer-reviewed publications using the China National Knowledge Infrastructure and Web of Science databases. The search term “*Spartina alterniflora*” was employed on both websites to compile a database of the *δ* ^13^C, *δ* ^15^N, and *δ* ^34^S values of *S. alterniflora*. We gathered 13540 published papers and dissertations from January 1970 to September 2023. Additionally, we identified relevant literature in Chinese or English related to the *δ* ^13^C, *δ* ^15^N, and *δ* ^34^S of *S. alterniflora* through manual screening methods. During the screening process, publications without latitude and longitude (or map, or location name), those with blurry images, or experiments conducted in greenhouses or involving isotope labeling processing about *δ* ^13^C, *δ* ^15^N, and *δ* ^34^S of *S*. *alterniflora* were excluded. After removing duplicates, the literature was refined to 57 publications, comprising 48 publications for *δ* ^13^C (1976–2023), 31 publications for *δ* ^15^N (1985–2022), and 8 publications for *δ* ^34^S (1982–2019) of *S. alterniflora* (**Figure 1**). The number of corresponding publications increased over time (**Supplementary Figure S1**).

### 2.2 Data extraction and proceeding

We extracted data from 57 publications using three methods: firstly, by recording the values of *δ* ^13^C, *δ* ^15^N, and *δ* ^34^S of *S. alterniflora* from tables; secondly, by measuring values from figures using Digitizer in Origin software; and thirdly, by collecting data from **Supplementary Materials** accompanying the publications. The criteria for data extraction included values for *δ* ^13^C, *δ* ^15^N, and *δ* ^34^S in various plant parts such as root (fine root, rhizome, coarse roots), stem (aboveground stem, belowground stem), leaf, litter (fresh litter, litter, leaf litter, plant detritus), standing dead, and dead biomass of *S. alterniflora*. Additionally, the criteria specified that the data should pertain to natural plants or samples collected in the field, be unlabeled with isotopes, or represent the initial values before isotope labeling. Furthermore, the values of isotope signatures were required to be expressed in parts per thousand (‰) and calculated following a specified equation (Equation 1).


(1) $$\delta X=\left[\left(R_{sample}/R_{standard}\right)\right]-1$$

Where *X* is ^13^C, ^15^N, or ^34^S, and *R* is ^13^C/^12^C, ^15^N/^14^N, ^34^S/^32^S, respectively (Wooller et al., 2003a; Gao et al., 2018; Nelson et al., 2019).

To make the information contained in the database more comprehensive, we recorded the following variables for each value: the first author, year of publication, study site, month or season in the year of sampling, latitude, longitude, treatment, plant tissue (root, stem, leaf), live or dead state, reference.

If the latitude and longitude information for the study sites was not available in the publication, we opted to identify them on the Ovital map, primarily using the map of sampling points and secondarily relying on the name of the study site. In specific cases, we distinguished between live *S. alterniflora* by examining green leaves and categorized plant senescent tissue as dead *S. alterniflora*.

**Figure 2:**
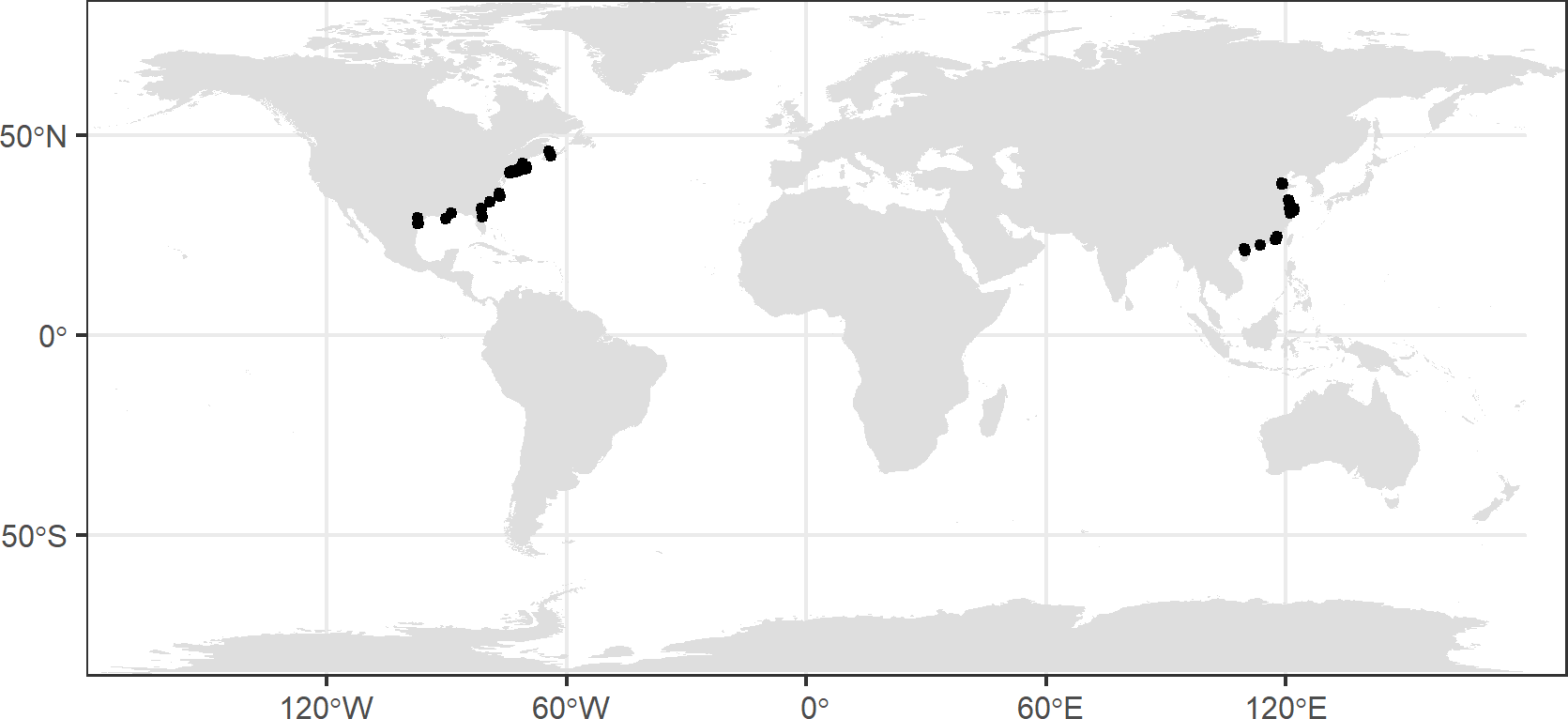
Global distribution of the sample sites of the isotope signature of *Spartina alterniflora.*.

We collected climate data for each sample site from the Worldclim online repository (https://www.worldclim.org/data/index.html; Fick & Hijmans, 2017). The mean annual temperature (MAT) and mean annual precipitation (MAP) were calculated based on the extracted data from Worldclim 2.1 referencing the latitude and longitude of sample sites (**Figure 2**).

### 2.3 Statistical analysis

The distribution and homogeneity of *δ* ^13^C, *δ* ^15^N, and *δ* ^34^S isotope signature (*δ*X) in *S. alterniflora* were assessed in R before conducting further analyses (**Supplementary Figure S2**). A t-test was employed to determine differences in the values of *δ* ^13^C, *δ* ^15^N, and *δ* ^34^S between live and dead *S. alterniflora* at a 0.05 significance level. Linear regression and binomial regression were utilized to explore the responses in the values of *δ* ^13^C, *δ* ^15^N, and *δ* ^34^S for the whole plant, live *S. alterniflora*, and dead *S. alterniflora* to latitude, MAT, and MAP using paired data (*δ*X-latitude, *δ*X-MAT, *δ*X-MAP). In cases where neither of the two regressions mentioned above matched, loess regression was applied to illustrate the changes in *δ* ^13^C, *δ* ^15^N, and *δ* ^34^S with increasing latitude, MAT, and MAP. Additionally, the relationship between any two indicators (paired data) of *δ* ^13^C, *δ* ^15^N, and *δ* ^34^S was examined using the previously mentioned regression techniques.

## 3 Results

### 3.1 Latitudinal patterns of the *δ* ^13^C, *δ* ^15^N, *δ* ^34^S in *S. alterniflora*

**Figure 3:**
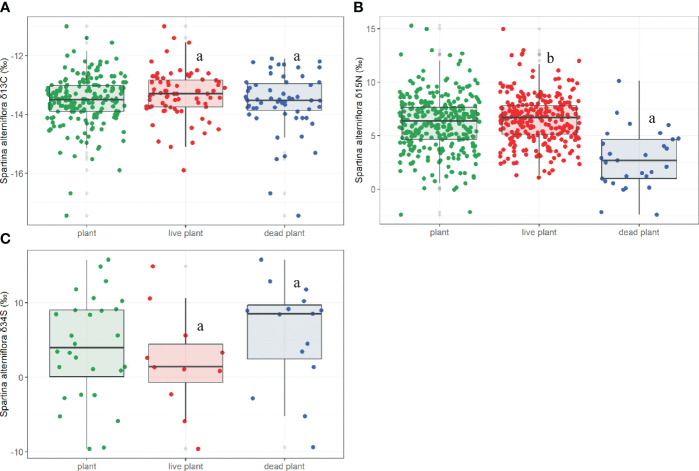
The *δ* ^13^C **(A)**, *δ* ^15^N **(B)**, *δ* ^34^S **(C)** in live and dead *S. alterniflora*. Different letters stand for significant differences at the 0.05 significance level in live and dead *S. alterniflora*.

**Figure 4:**
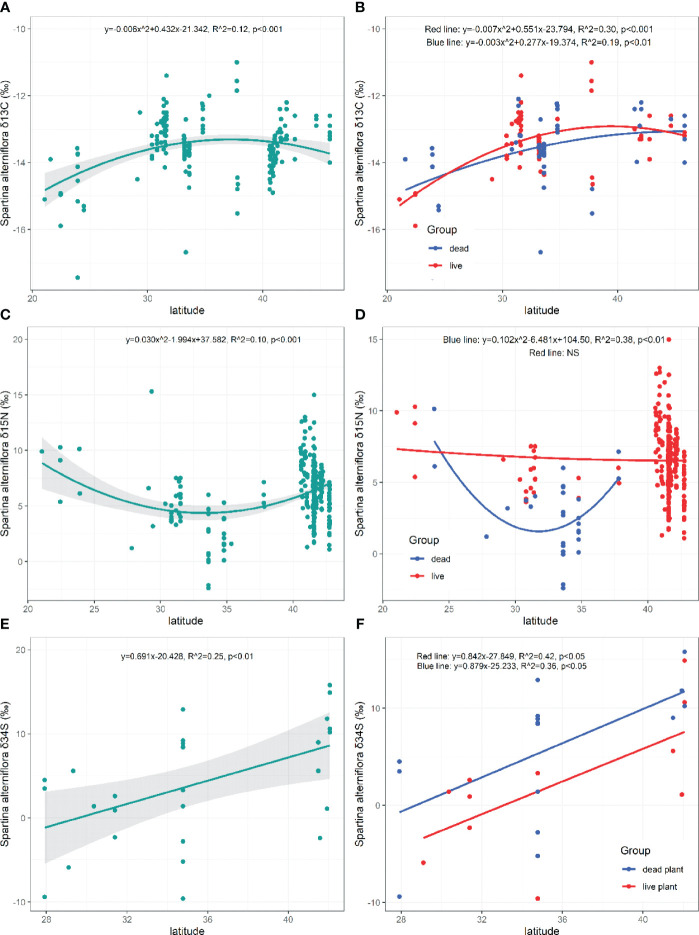
Latitudinal patterns of the of *δ* ^13^C, *δ* ^15^N, *δ* ^34^S in live and dead *S. alterniflora*. NS, the fitting is not statistically significant.

The value of *δ* ^13^C of *S. alterniflora* ranged from -17.44‰ to -11.00‰ (*M*=-13.52 ± 0.83‰, *N*=195; **Figure 3A**) and firstly increased and then decreased with increasing latitudes (*p*=0.000, **Figure 4A**).

Both the *δ* ^13^C in live and dead *S. alterniflora* displayed similar patterns toward higher latitudes (Live: *p*=0.000; Dead: *p*=0.003, **Figure 4B**). There were no significant differences in the *δ* ^13^C of live (*M*=-13.32 ± 0.82‰, *N*=72) and dead *S. alterniflora* (*M*=-13.59 ± 1.01‰, *N*=59; *p*=0.097; **Figure 3A**). The *δ* ^15^N values of *S. alterniflora* ranged from -2.40‰ to 15.30‰ (*M*=6.16 ± 0.14‰, *N*=316; **Figure 3B**) and initially decreased and then increased with increasing latitudes (*p*=0.000, **Figure 4C**). The *δ* ^15^N of dead *S. alterniflora* displayed similar patterns toward higher latitudes, unlike the live *S. alterniflora* (Live: *p*=0.756, Dead: *p*=0.002; **Figure 4D**). The *δ* ^15^N of live *S. alterniflora* (*M*=6.55 ± 2.23‰, *N*=272) was significantly higher than that of dead *S. alterniflora* (*M*=-2.76 ± 2.72‰, *N*=29; *p*=0.000; **Figure 3B**). The *δ* ^34^S values of *S. alterniflora* ranged from -9.60‰ to 15.80‰ (*M*=4.01 ± 6.96‰, *N*=28; **Figure 3C**) and showed a significant increase toward higher latitudes (*p*=0.006, **Figure 4E**). Both the *δ* ^34^S in live and dead *S. alterniflora* displayed similar patterns toward higher latitudes (Live: *p*=0.030; Dead: *p*=0.016, **Figure 4F**), but live *S. alterniflora* had a higher fitting value of *δ* ^34^S at the same latitude. There were no significant differences in the *δ* ^34^S of live (*M*=2.05 ± 6.88‰, *N*=11) and dead *S. alterniflora* (*M*=5.78 ± 7.09‰, *N*=15; *p*=0.193; **Figure 3C**).

### 3.2 Responses of the *δ* ^13^C, *δ* ^15^N, *δ* ^34^S in *S. alterniflora* to MAT and MAP

**Figure 5:**
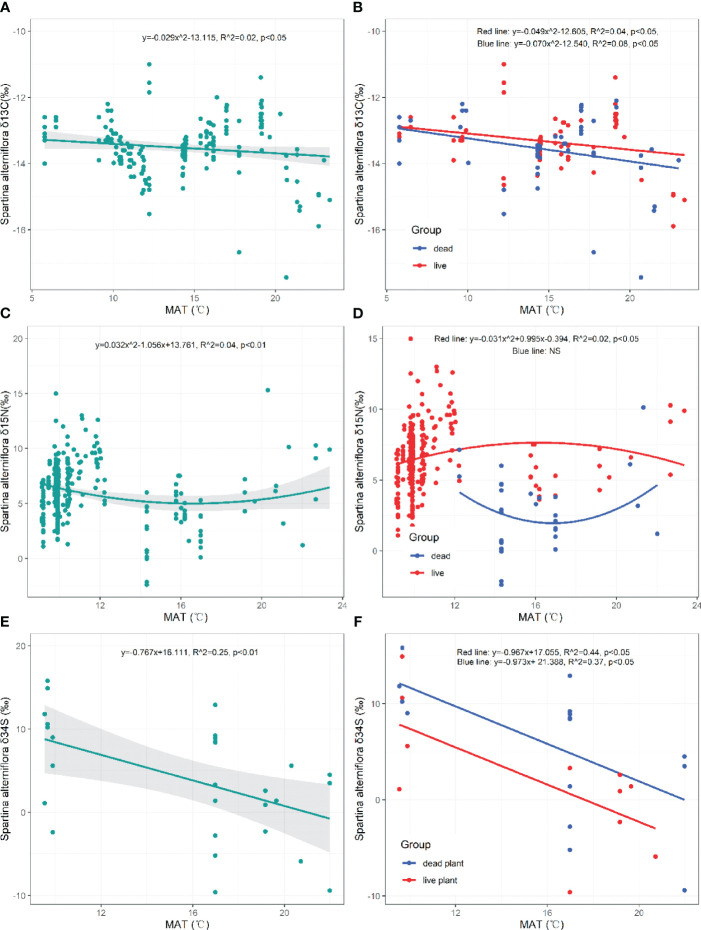
Responses of *δ* ^13^C, *δ* ^15^N, *δ* ^34^S in live and dead *S. alterniflora* to mean annual temperature (MAT). NS, the fitting is not statistically significant.

The *δ* ^13^C value of *S. alterniflora* significantly decreased with higher MAT (*p*=0.050, **Figure 5A**). Both the *δ* ^13^C values of live and dead *S. alterniflora* showed significant decreasing trends at elevated MAT levels (Live: *p*=0.029, Dead: *p*=0.026, **Figure 5B**). The *δ* ^15^N value of *S. alterniflora* initially declined and then increased with rising MAT (*p*=0.002, **Figure 5C**). However, the *δ* ^15^N value of live *S. alterniflora* first increased and then decreased in response to MAT (Live: *p*=0.043, **Figure 5D**). The *δ* ^34^S value of *S. alterniflora* (both live and dead) exhibited a significant decrease with increasing MAT (Plant: *p*=0.007, Live: *p*=0.025, Dead: *p*=0.016, **Figures 5E, F**).

**Figure 6:**
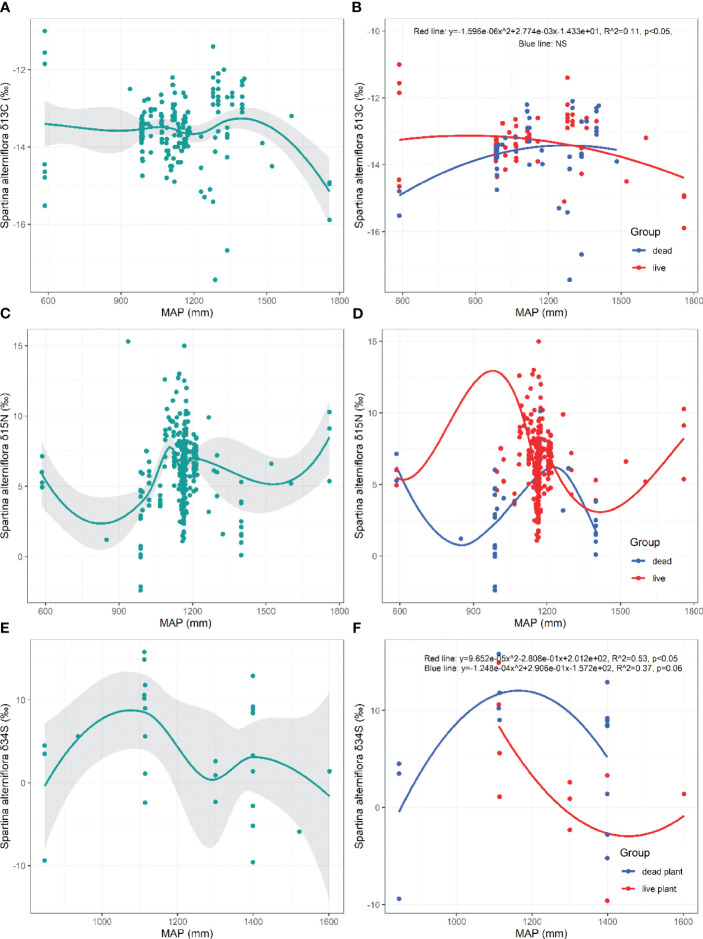
Responses of *δ* ^13^C, *δ* ^15^N, *δ* ^34^S in live and dead *S. alterniflora* to mean annual precipitation (MAP). NS, the fitting is not statistically significant.

There are no significant trends in the *δ* ^13^C, *δ* ^15^N, and *δ* ^34^S values of *S. alterniflora* with increasing MAP (*p*>0.05, **Figures 6A, C–E**). The *δ* ^13^C of live *S*. *alterniflora* exhibited a significant decreasing trend when the MAP is >869 mm (*p*=0.017, **Figure 6B**). The *δ* ^34^S value of live *S. alterniflora* initially decreased and then increased with increasing MAP (Live: *p*=0.048, **Figure 6F**), whereas the *δ* ^34^S of dead *S. alterniflora* initially increased and then decreased with increasing MAP (Dead: *p*=0.063, **Figure 6F**).

### 3.3 Relationships of the *δ* ^13^C, *δ* ^15^N, *δ* ^34^S in *S. alterniflora*

**Figure 7:**
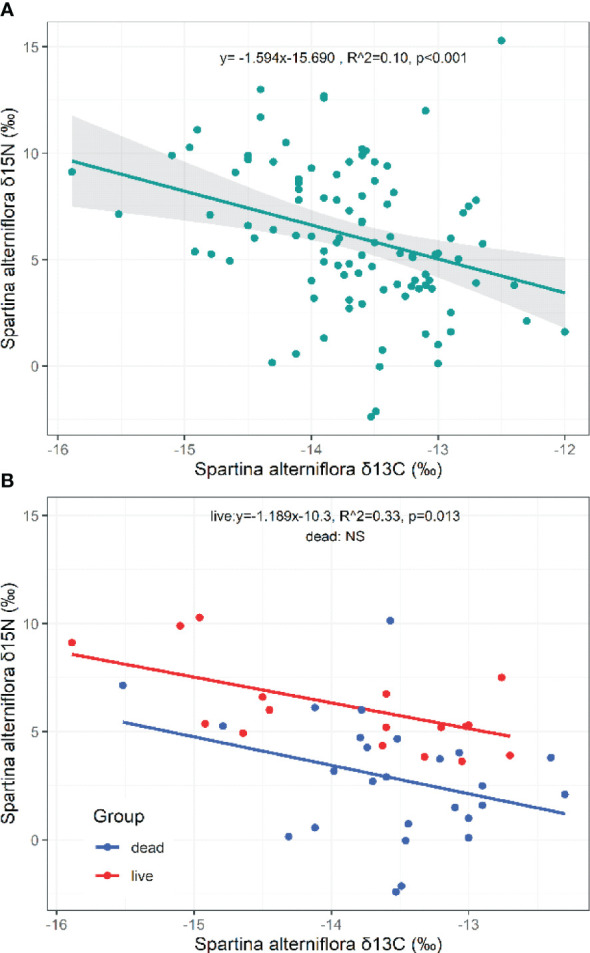
Relationships of the *δ* ^13^C and *δ* ^15^N in *S. alterniflora*. NS, the fitting is not statistically significant.

The *δ* ^15^N of *S. alterniflora* showed a significant negative relationship with the *δ* ^13^C of *S. alterniflora* (*p*=0.001, *N*=102; **Figure 7A**). Plant life status shaped a decreasing curve for the *δ* ^15^N in response to increasing *δ* ^13^C of *S. alterniflora* (Live: *p*=0.013, *N*=18, **Figure 7B**). However, the relationships between the *δ* ^34^S and *δ* ^13^C of *S. alterniflora* (*p*=0.110, *N*=14; **Supplementary Figure S3**), the *δ* ^34^S and *δ* ^15^N (*p*=0.667, *N*=14; **Supplementary Figure S3**) of *S. alterniflora* (or dead or live) were fuzzy.

## 4 Discussion

We observed a consistent pattern in the *δ* ^13^C, *δ* ^15^N, and *δ* ^34^S values of *S. alterniflora* across latitudes, providing evidence for the latitudinal variation in vegetative growth traits. These traits are likely associated with the plant’s invasive mechanisms. Previous studies have documented general patterns, such as linear or binomial, in plant height, stem diameter, leaf characteristics (leaf area, thickness, toughness, specific leaf area, dry matter content), shoot density, plant productivity, reproductive traits (seed set, seed production, seeding density, seed germination, first flower day, flowering culm, number of spikelets), ecostoichiometry (C, N, P, and their ratios), and leaf litter decomposition rate in response to increasing latitudes within the specified range (Kirwan et al., 2009; Liu et al., 2017; Liu et al., 2020; Liu et al., 2020; Chen et al., 2022; Cheng et al., 2022; Liu et al., 2022; Zhang et al., 2021a; Zheng et al., 2022). The latitudinal patterns of vegetative traits collectively regulate the growth, reproduction, and expansion of *S. alterniflora* through plastic deformation strategies. The plastic deformation strategies altered plant form and function so as to match environmental changes in a new environment, these enhancing plants competitiveness for space and resources, allowing it to occupy vacant ecological niches (Liu et al., 2017; Chen et al., 2023; Xiong et al., 2023). Previous research has shown that the plant height of *S. alterniflora* forms a convex curve with increasing latitudes (20°N~40°N), aligning with the latitudinal pattern of *δ* ^13^C but contrasting with that of *δ* ^15^N in *S*. *alterniflora* (Liu et al., 2017). In general, larger *S. alterniflora* plants with greater height exhibit strong photosynthesis, altering carbon isotope fractionation and increasing the potential accumulation of ^13^C (Lin and da SL Sternberg, 1993; Essemine et al., 2020; Mao et al., 2023). When nutrient resources are limited, larger plants typically require a significant amount of nutrients to sustain rapid growth, resulting in a dilution of ^15^N and a decrease in its accumulation within the plant (Hill et al., 2018; Chen et al., 2022). Moreover, an increase in dry matter content induces plant senescence, leading to a reduction in ^15^N-related enzymes and influencing plant ^15^N levels (Hessini, 2022; Xia et al., 2023a). Hence, the varied latitudinal patterns of *S. alterniflora*’s plant traits facilitate its adaptive response to environmental changes associated with different latitudes.

Plant life-death status have complex effects on isotopes, and then affecting their latitudinal patterns. In this study, the plant life status did not alter the *δ* ^13^C and *δ* ^34^S values or their corresponding latitudinal patterns in *S. alterniflora*. These findings are consistent with prior research; Schwamborn et al. (2002) and Kieckbusch et al. (2004) discovered that live leaves and senescent leaves exhibited similar *δ* ^13^C values. Similar evidence was observed in the *δ* ^13^C of *Kandelia candel* and *Rhiziophora mangle* during the senescence process (Wooller et al., 2003b). However, Rao et al. (1994) reported no difference in *δ* ^13^C between fresh and senescent tissues of five mangrove species in Kenya, while the other four mangrove species displayed differentiation. Robin et al. (2024) found that fresh leaves are more enriched in ^13^C than senescent leaves for *Avicennia marina* and *Rhizophora stylosa*, but for *R. stylosa* they are less enriched in ^15^N. Furthermore, we observed that the death of plants led to a reduction in *δ* ^15^N in *S. alterniflora* compared to their living status. This phenomenon may be attributed to the inactivation of N-related enzymes in *S. alterniflora* (Hessini, 2022; Xia et al., 2023a). The status of plant death resulted in a *δ* ^15^N response in the form of a concave curve with increasing latitude but exhibited a less distinct response under the plant life status. These findings suggest that live *S. alterniflora* demonstrates a broad range of *δ* ^15^N responses to latitude, particularly at higher latitudes. There is no consensus on whether there are differences in plant isotopes and their latitudinal patterns across species under different life states. This inconsistency with our hypothesis (1) leads us to speculate that variations in plant isotopes and their latitudinal patterns under different life status may depend on the species, its strength, the type of isotopes (Wooller et al., 2003b; Rao et al., 1994), and specific evidence that needs further exploration.

MAT and MAP play crucial roles in shaping latitude and influencing plant growth and reproduction (Yuan and Chen, 2009; Zhang et al., 2019; Xia et al., 2023a). Prior studies have demonstrated that temperature and precipitation contribute to the formation of linear or quadratic polynomial patterns in plant growth and reproductive traits (Kirwan et al., 2009; Liu et al., 2017, Liu et al., 2020; Liu et al., 2020; Liu et al., 2022; Chen et al., 2022; Cheng et al., 2022; Zheng et al., 2022). Latitude changes increase environmental heterogeneity, especially in hydrothermal environments; Simultaneously, it shapes different plant traits to adapt to environmental changes. Therefore, we hypothesize that the isotopes of *S. alterniflora*, closely linked to these traits, are affected by MAT and MAP. While general patterns of *δ* ^13^C, *δ* ^15^N, and *δ* ^34^S in response to MAT along latitudinal gradients were identified, this was not observed for MAP, which is not entirely consistent with our hypothesis (2). A consensus emerges, indicating that MAT is the controlling factor for the latitudinal patterns of *δ* ^13^C, *δ* ^15^N, and *δ* ^34^S in *S. alterniflora*. The marginal influence of precipitation in this study warrants thorough consideration, deviating from previous research conclusions (Yuan and Chen, 2009; Xia et al., 2023a). In **Supplementary Figure S2**, MAP exhibits a trend of decreasing and then increasing with latitude. Additionally, MAP is influenced by various factors, including the relative position of land and sea, terrain, pressure bands, wind belts, monsoons, cyclones, fronts, underlying surfaces, ocean currents, and human activities (Fick & Hijmans, 2017). The complexity of these factors reduces the interpretability of latitude’s impact on MAP and disrupts the response patterns of *δ* ^13^C, *δ* ^15^N, and *δ* ^34^S in *S. alterniflora* to MAP. Interestingly, the *δ* ^15^N of *S. alterniflora* under live plant conditions shows a significant response to MAT, unlike its death status. This observation aligns with the notion that temperature influences enzyme activity in live plant bodies (Hessini, 2022). The *δ* ^13^C and *δ* ^34^S of live *S. alterniflora* exhibit a significant response to MAP due to their stability and the importance of precipitation for plant survival (Yuan and Chen, 2009; Xia et al., 2023a). Phenotypic plasticity, by altering plant isotopes and their other traits, fully ensures their survival in changing environments, and enables invasive plants to successfully customize and spread in the invasive ranges.

The relationships among plant elements or isotopes are crucial for studying plant adaptation mechanisms at the single-species scale and element flux in food webs at the whole ecosystem scale (Gao et al., 2018; Jinks et al., 2020; Li et al., 2022). However, the paired relationships between any two indicators of *δ* ^13^C, *δ* ^15^N, and *δ* ^34^S in *S. alterniflora* have been overlooked. In this study, we hypothesize that the *δ* ^15^N and *δ* ^34^S of *S. alterniflora* exhibit stable relationships with the corresponding *δ* ^13^C. The *δ* ^15^N of *S. alterniflora* decreases with increasing *δ* ^13^C, supporting parts of hypothesis (3). When having similar *δ* ^13^C values, the decrease in *δ* ^15^N of deceased *S. alterniflora* disrupts the general relationship between *δ* ^13^C and *δ* ^15^N. Additionally, the indistinct relationships between *δ* ^34^S and *δ* ^13^C (or *δ* ^15^N) of *S. alterniflora* may be associated with the *δ* ^34^S in the environment. Excessive *δ* ^34^S in the soil (found in salt marshes with sulfate-type soil) or atmosphere (in areas with acid rain) leads to an increase in *δ* ^34^S in plant bodies, resulting in a mismatch in the relationship between *δ* ^34^S and *δ* ^13^C (or *δ* ^15^N) of *S. alterniflora* (Guo et al., 2020; Jinks et al., 2020; Guiry et al., 2022). These findings highlight the isotopic flexibility within *S. alterniflora*.

Although our results provide strong evidence for the latitudinal patterns and their climate drivers of the *δ* ^13^C, *δ* ^15^N, *δ* ^34^S isotope signatures of *S. alterniflora* across plant life-death status based on a global analysis, there were several limitations in this study, such as how soil isotope signatures affected plant isotope signatures. Soil is recognized as a key factor influencing available nutrient for plant, and their large-scale patterns of isotope signatures have an imprint on plant isotope signatures (Amundson et al., 2003; Craine et al., 2015). Plant *δ* ^13^C (*δ* ^15^N) was significantly positively related to soil *δ* ^13^C (*δ* ^15^N) across varied plant species and functional types (Peri et al., 2012; Xia et al., 2023b). Although soil *δ* ^13^C and *δ* ^15^N was shaped by MAT and MAP, future research should pay attentions to the imprint of soil isotope signatures on corresponding isotope signatures.

## 5 Conclusions

The latitudinal patterns of *δ* ^13^C, *δ* ^15^N, and *δ* ^34^S in *S. alterniflora* are depicted as a convex curve, a concave curve, and an increasing straight line, respectively. The responses of *δ* ^13^C, *δ* ^15^N, and *δ* ^34^S in *S. alterniflora* to MAT manifest as a decreasing line, a concave curve, and a decreasing line, respectively, but exhibit indistinct responses to MAP. The life-death status of plants alters the *δ* ^15^N-latitudinal patterns and their responses of *δ* ^13^C, *δ* ^15^N, and *δ* ^34^S in *S. alterniflora* to MAT. The *δ* ^13^C and *δ* ^34^S of living *S. alterniflora* demonstrate robust responses to MAP. Plant death status results in a significant decrease in *δ* ^15^N, but not in *δ* ^13^C and *δ* ^34^S. Paired *δ* ^15^N and *δ* ^13^C of *S. alterniflora* exhibit a noteworthy negative relationship across the entire dataset and plant life status. All these findings provide evidence of robust ecological plasticity and adaptability across geographic clines.

## Data availability statement

The raw data supporting the conclusions of this article will be made available by the authors, without undue reservation.

## Author contributions

DZ: Writing – original draft, Writing – review & editing. HW: Data curation, Investigation, Visualization, Writing – original draft. XL: Data curation, Investigation, Writing – original draft. KA: Data curation, Writing – original draft. WH: Writing – review & editing. TW: Data curation, Investigation, Writing – original draft. MZ: Writing – original draft, Writing – review & editing. ST: Conceptualization, Writing – review & editing.
