# Supplementary material for: Latitudinal patterns and their climate drivers of the δ 13C, δ 15N, δ 34S isotope signatures of Spartina alterniflora across plant life-death status: a global analysis
Source: Front Plant Sci. 2024 May 31;15:1384914. doi: 10.3389/fpls.2024.1384914 (PMC11176468; doi:10.3389/fpls.2024.1384914)
Supplement: Supplementary file 1 [file DataSheet_1.docx]

**Supplementary materials**

Figure.S1 Number of relevant studies included in this study (*N*=57) published per year (1978-2023)


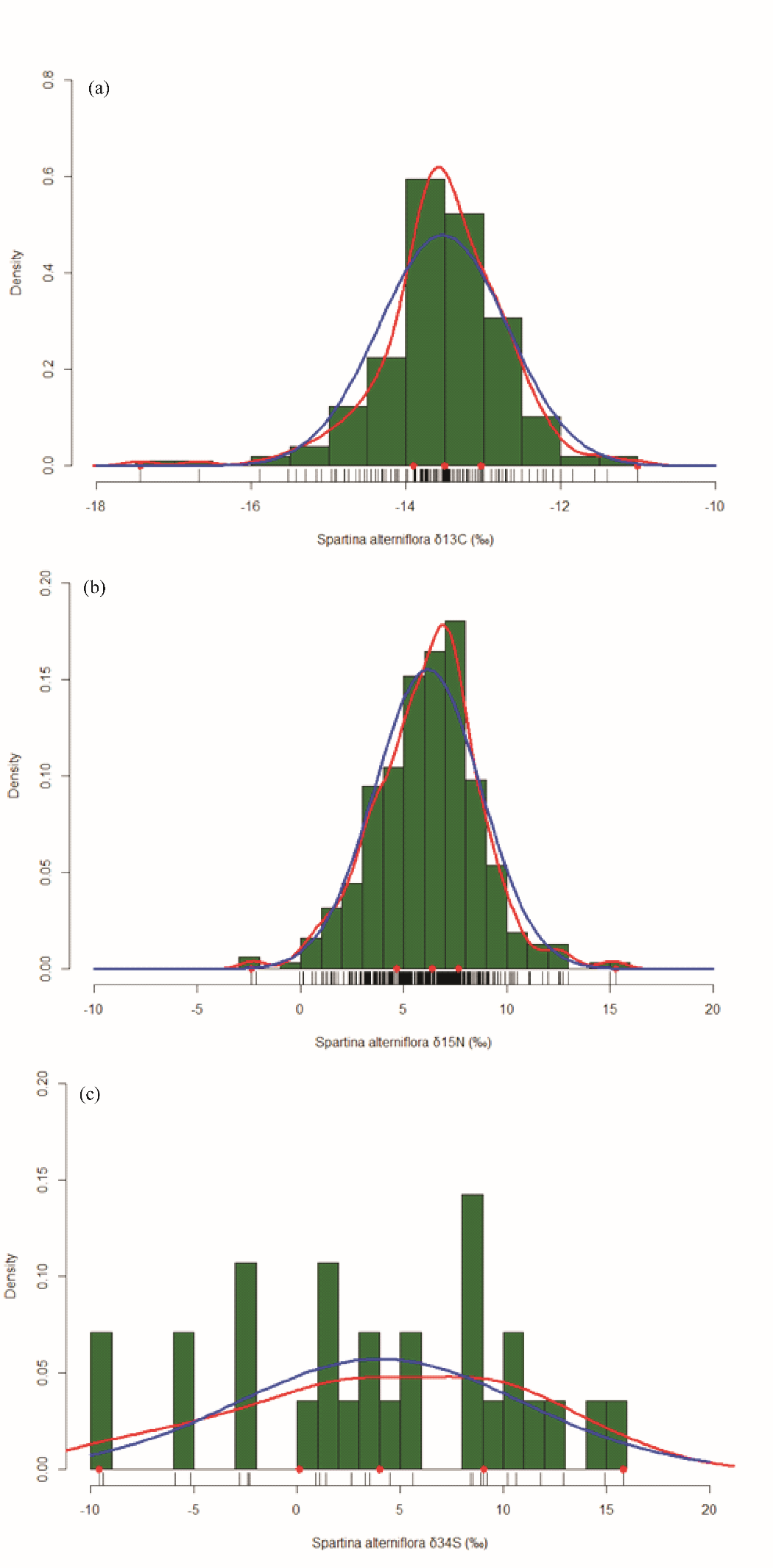


Figure.S2 Data distribution of the isotope signature of *Spartina alterniflora*


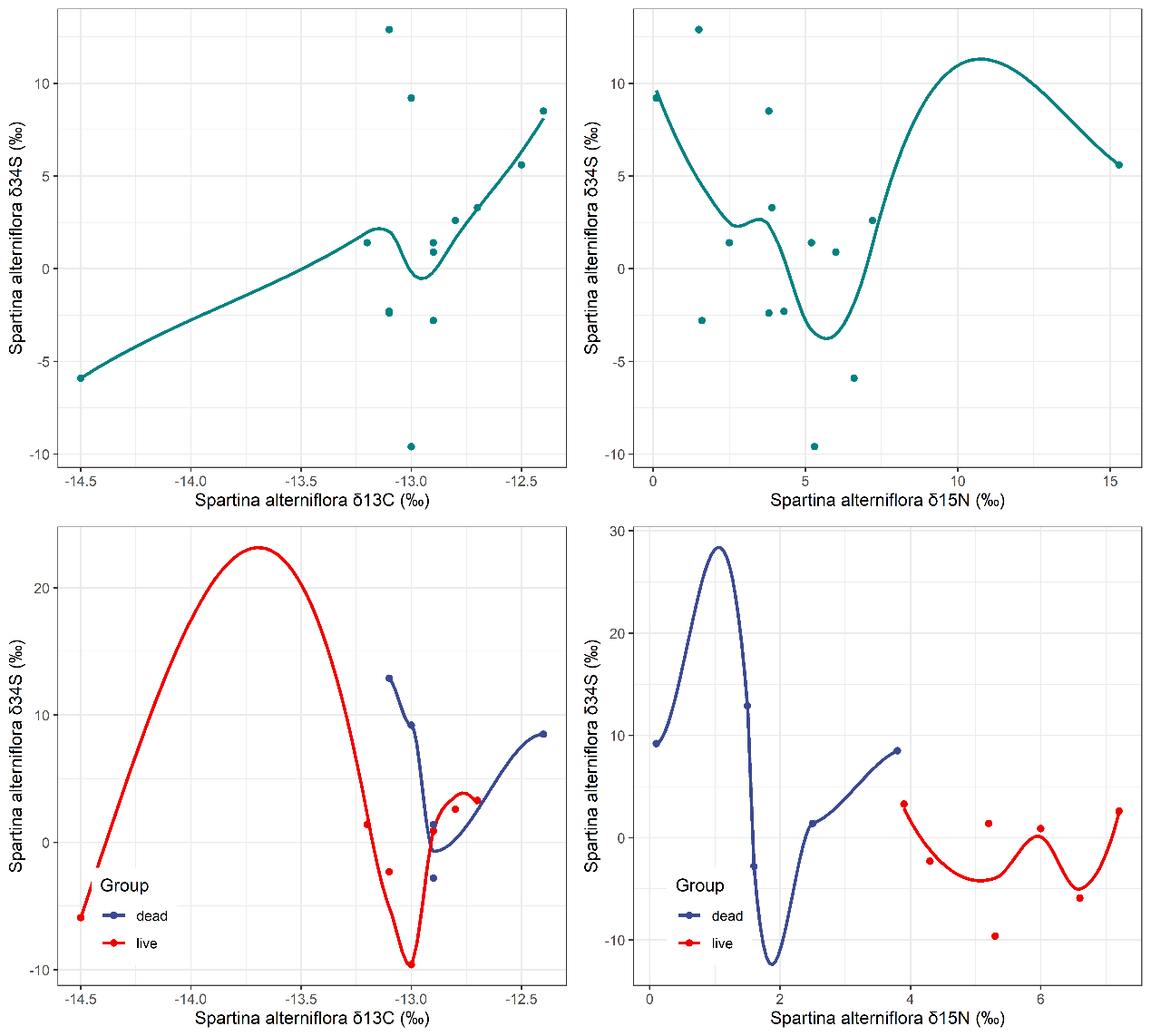


(d)

(c)

(b)

(a)


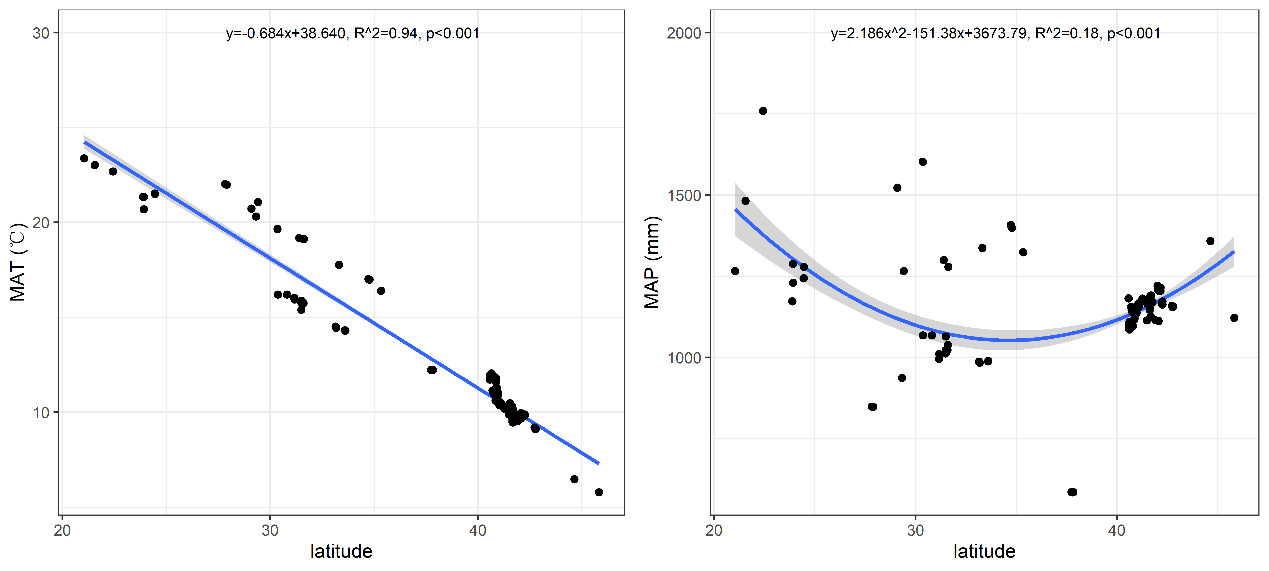
Figure.S3 Relationships of the *δ*^34^S vs *δ*^13^C, *δ*^34^S vs *δ*^15^N in *S. alterniflora*

(b)

(a)

Figure.S4 Latitudinal patterns of the mean annual temperature (MAT) and mean annual precipitation (MAP) in sample sites
