# Supplementary material for: Latitudinal patterns and their climate drivers of the δ 13C, δ 15N, δ 34S isotope signatures of Spartina alterniflora across plant life-death status: a global analysis
Source: Front Plant Sci. 2024 May 31;15:1384914. doi: 10.3389/fpls.2024.1384914 (PMC11176468; doi:10.3389/fpls.2024.1384914)
Supplement: Supplementary file 2 [file DataSheet_2.docx]

**List of 57 publications related to the data of the *δ*^13^C, *δ*^15^N, *δ*^34^S of *S. alterniflora***

1. Zhang, G., Bai, J., Zhao, Q., Jia, J., Wang, X., Wang, W., & Wang, X. (2021). Soil carbon storage and carbon sources under different Spartina alterniflora invasion periods in a salt marsh ecosystem. Catena, 196, 104831.
2. Nelson, J. A., Lesser, J., James, W. R., Behringer, D. P., Furka, V., & Doerr, J. C. (2019). Food web response to foundation species change in a coastal ecosystem. Food Webs, 21, e00125.
3. Alberts, J. J., Filip, Z., Price, M. T., Williams, D. J., & Williams, M. C. (1988). Elemental composition, stable carbon isotope ratios and spectrophotometric properties of humic substances occurring in a salt marsh estuary. Organic Geochemistry, 12(5), 455-467.
4. Chen, H., Feng, J., Zhang, Y., Wei, S., Chen, Z., & Lin, G. (2021). Significant but short time assimilation of organic matter from decomposed exotic Spartina alterniflora leaf litter by mangrove polychaetes. Estuarine, Coastal and Shelf Science, 259, 107436.
5. Cheng, X., Luo, Y., Chen, J., Lin, G., Chen, J., & Li, B. (2006). Short-term C4 plant Spartina alterniflora invasions change the soil carbon in C3 plant-dominated tidal wetlands on a growing estuarine Island. Soil Biology and Biochemistry, 38(12), 3380-3386.
6. Gao, X., Wang, M., Wu, H., Wang, W., & Tu, Z. (2018). Effects of Spartina alterniflora invasion on the diet of mangrove crabs (*Parasesarma plicata*) in the Zhangjiang Estuary, China. Journal of Coastal Research, 34(1), 106-113.
7. Gao, J., Bai, F., Yang, Y., Gao, S., Liu, Z., & Li, J. (2012). Influence of Spartina colonization on the supply and accumulation of organic carbon in tidal salt marshes of northern Jiangsu Province, China. Journal of Coastal Research, 28(2), 486-498.
8. Ju, R. T., Chen, Y. Y., Gao, L., & Li, B. (2016). The extended phenology of Spartina invasion alters a native herbivorous insect’s abundance and diet in a Chinese salt marsh. Biological invasions, 18(8), 2229-2236.
9. Watson, E. B., Powell, E., Maher, N. P., Oczkowski, A. J., Paudel, B., Starke, A., ... & Wigand, C. (2018). Indicators of nutrient pollution in Long Island, New York, estuarine environments. Marine environmental research, 134, 109-120.
10. Currin, C. A., Newell, S. Y., & Paerl, H. W. (1995). The role of standing dead Spartina alterniflora and benthic microalgae in salt marsh food webs: considerations based on multiple stable isotope analysis. Marine Ecology Progress Series, 121, 99-116.
11. Couch, C. A. (1989). Carbon and nitrogen stable isotopes of meiobenthos and their food resources. Estuarine, Coastal and Shelf Science, 28(4), 433-441.
12. Ember, L. M., Williams, D. F., & Morris, J. T. (1987). Processes that influence carbon isotope variations in salt marsh sediments. Marine Ecology Progress Series, 36(1), 33-42.
13. Fogel, M. L., Sprague, E. K., Gize, A. P., & Frey, R. W. (1989). Diagenesis of organic matter in Georgia salt marshes. Estuarine, Coastal and Shelf Science, 28(2), 211-230.
14. Haddad, R. I., Newell, S. Y., Martens, C. S., & Fallon, R. D. (1992). Early diagenesis of lignin-associated phenolics in the salt marsh grass Spartina alterniflora. Geochimica et Cosmochimica Acta, 56(10), 3751-3764.
15. Schwinghamer, P., Tan, F. C., & Gordon Jr, D. C. (1983). Stable carbon isotope studies on the Pecks Cove mudflat ecosystem in the Cumberland Basin, Bay of Fundy. Canadian Journal of Fisheries and Aquatic Sciences, 40(S1), s262-s272.
16. Stephenson, R. L., Tan, F. C., & Mann, K. H. (1986). Use of stable carbon isotope ratios to compare plant material and potential consumers in a seagrass bed and a kelp bed in Nova Scotia, Canada. Marine Ecology Progress Series, 1-7.
17. Haines, E. B. (1976). Stable carbon isotope ratios in the biota, soils and tidal water of a Georgia salt marsh. Estuarine and Coastal Marine Science, 4(6), 609-616.
18. Craft, C. B., Broome, S. W., Seneca, E. D., & Showers, W. J. (1988). Estimating sources of soil organic matter in natural and transplanted estuarine marshes using stable isotopes of carbon and nitrogen. Estuarine, Coastal and Shelf Science, 26(6), 633-641.
19. Deegan, L. A., Peterson, B. J., & Portier, R. (1990). Stable isotopes and cellulase activity as evidence for detritus as a food source for juvenile Gulf menhaden. Estuaries, 13(1), 14-19.
20. Sullivan, M. J., & Moncreiff, C. A. (1990). Edaphic algae are an important component of salt marsh food-webs: evidence from multiple stable isotope analyses. Marine ecology progress series. Oldendorf, 62(1), 149-159.
21. Peterson, B. J., & Howarth, R. W. (1987). Sulfur, carbon, and nitrogen isotopes used to trace organic matter flow in the salt‐marsh estuaries of Sapelo Island, Georgia 1. Limnology and oceanography, 32(6), 1195-1213.
22. Peterson, B. J., Howarth, R. W., & Garritt, R. H. (1985). Multiple stable isotopes used to trace the flow of organic matter in estuarine food webs. Science, 227(4692), 1361-1363.
23. Miao, S. Y., Long, L. D., Tao, W. Q., Zeng, Q. C., Chen, J. H., Huang, J. L., ... & Tang, Y. J. (2016). Composition of stable carbon and nitrogen isotopes in five wetland plants and sediments from the Pearl River estuary, South China. Chemistry and Ecology, 32(7), 609-623.
24. Wang, M., Wang, Q., Sha, C., & Chen, J. (2018). Spartina alterniflora invasion affects soil carbon in a C3 plant-dominated tidal marsh. Scientific Reports, 8(1), 628.
25. Simpson, L. T., Cherry, J. A., Smith, R. S., & Feller, I. C. (2021). Mangrove encroachment alters decomposition rate in saltmarsh through changes in litter quality. Ecosystems, 24, 840-854.
26. Wang, X. C., Chen, R. F., & Berry, A. (2003). Sources and preservation of organic matter in Plum Island salt marsh sediments (MA, USA): long-chain n-alkanes and stable carbon isotope compositions. Estuarine, Coastal and Shelf Science, 58(4), 917-928.
27. Wang, S., He, Q., Zhang, Y., Sheng, Q., Li, B., & Wu, J. (2021). Habitat‐dependent impacts of exotic plant invasions on benthic food webs in a coastal wetland. Limnology and Oceanography, 66(4), 1256-1267.
28. Wozniak, A. S., Roman, C. T., Wainright, S. C., McKinney, R. A., & James-Pirri, M. J. (2006). Monitoring food web changes in tide-restored salt marshes: a carbon stable isotope approach. Estuaries and Coasts, 29, 568-578.
29. Yan, J., Wang, L., Hu, Y., Tsang, Y. F., Zhang, Y., Wu, J., ... & Sun, Y. (2018). Plant litter composition selects different soil microbial structures and in turn drives different litter decomposition pattern and soil carbon sequestration capability. Geoderma, 319, 194-203.
30. Yang, W., An, S., Zhao, H., Fang, S., Xia, L., Xiao, Y., ... & Cheng, X. (2015). Labile and Recalcitrant Soil Carbon and Nitrogen Pools in Tidal Salt Marshes of the Eastern Chinese Coast as Affected by Short‐Term C4 Plant Spartina alterniflora Invasion. Clean–Soil, Air, Water, 43(6), 872-880.
31. Zhang, Y., Ding, W., Luo, J., & Donnison, A. (2010). Changes in soil organic carbon dynamics in an Eastern Chinese coastal wetland following invasion by a C4 plant Spartina alterniflora. Soil Biology and Biochemistry, 42(10), 1712-1720.
32. Zhang, Y., Ding, W., Luo, J., & Donnison, A. (2010). Changes in soil organic carbon dynamics in an Eastern Chinese coastal wetland following invasion by a C4 plant Spartina alterniflora. Soil Biology and Biochemistry, 42(10), 1712-1720.
33. Jiang, J.J., Zhao, Y.J., Zhao, J.Y., et al. Restoration of diets of herbivorous insects following the removal of invasive *Spartina alterniflora* in a saltmarsh [J]. Journal of Fudan University (NaturalScience), 2021, 60(4): 444-450. (in Chinese, 江佳佳, 赵玉杰, 赵佳媛等. 盐沼入侵植物互花米草治理后植食性昆虫食物结构的变化[J]. 复旦学报(自然科学版), 2021, 60(4): 444-450.)
34. Wu, Y. Decomposition dynamics of organic carbon in *Spartina alterniflora* litter in situ [D]. Nanjing Normal University, 2016. (in Chinese, 吴亚萍. 互花米草(*Spartina alterniflora*)凋落物有机碳原位分解动态[D].南京师范大学, 2016.)
35. Wang, W. Nutritional dynamics and stable isotope indications in tidal flats of the Yangtze Estuary [D]. East China Normal University, 2011. (in Chinese, 王伟伟. 长江口潮滩营养动态与稳定同位素指示研究[D]. 华东师范大学, 2011.)
36. Feng, Z. The impact of biomass change of *Spartina alterniflora* on the organic carbon content in salt marsh, a case study of tidal salt marsh of Wanggang Estuary, Jiangsu Province [D]. Nanjing University, 2015. (in Chinese, 王伟伟. 长江口潮滩营养动态与稳定同位素指示研究[D]. 华东师范大学, 2011.)
37. Jiang, S., Chen, L., Yan, L., et al. Impacts of *Spartina alterniflora* invasion on the benthic food web in the Yellow River Delta during autumn. Chinese Journal of Applied Ecology, 2021, 32(12): 4499-4507. (in Chinese, 姜少玉, 陈琳琳, 闫朗等. 互花米草入侵对黄河三角洲秋季底栖食物网的影响[J]. 应用生态学报, 2021, 32(12): 4499-4507. )
38. Yang, Y. Carbon, nitrogen and phosphorus stoichiometries of soil, microbial biomass, and extracellular enzyme activities across a transect in the tidal wetland of the Jiulong River Estuary, China[D]. Fujian Normal University, 2022. (in Chinese, 杨洋. 九龙江河口潮滩剖面土壤、微生物量和胞外酶活性的碳氮磷计量比研究[D]. 福建师范大学, 2022. )
39. Peng, B., Wang, S., Zhao, F., et al. (2022). Carbon and nitrogen isotopic characteristics and food source differences of Lateolabrax maculatus in three sub habitats of salt marsh wetland in the Yangtze River Estuary [J]. Journal of Fishery Sciences of China, 29(2):295-303. (in Chinese, 彭彪彪, 王思凯, 赵峰等. 长江口盐沼湿地3种亚生境中国花鲈的碳、氮稳定同位素特征及基础食源差异[J]. 中国水产科学, 2022, 29(2): 295-303.
40. He, S., Lin, J., Liu, X., Jia, S., & Chen, S. (2023). Cordgrass Spartina alterniflora acts as a key carbon source to support macrozoobenthos in the salt marsh and nearby mudflat communities. Ecological Indicators, 148, 110052.
41. Wang, F., Sun, X., Zhao, Y., Wang, H., Song, X., Wei, S., & Chen, H. (2023). Does Spartina invasion affect the carbohydrate assimilation of polychaetes in mangroves? A case study in the Zhangjiang Estuary Mangrove National Nature Reserve. Journal of Sea Research, 195, 102435.
42. Zhu, P., Chen, X., Zhang, Y., Zhang, Q., Wu, X., Zhao, H., ... & Li, L. (2022). Porewater-derived blue carbon outwelling and greenhouse gas emissions in a subtropical multi-species saltmarsh. Frontiers in Marine Science, 9, 884951.
43. Li, X., Yang, W., Sun, T., & Yang, Z. (2022). Trophic diversity and food web structure of vegetated habitats along a coastal topographic gradient. Frontiers in Marine Science, 9, 920745.
44. Lu, Z., Xiao, K., Wang, F., Wang, Y., Yu, Q., & Chen, N. (2023). Salt marsh invasion reduces recalcitrant organic carbon pool while increases lateral export of dissolved inorganic carbon in a subtropical mangrove wetland. Geoderma, 437, 116573.
45. Zhang, Z., & Chen, S. (2022). Carbon sources and trophic levels in the food web of the largest mangrove reserve of China. Continental Shelf Research, 248, 104854.
46. Chen, H., Feng, J., Zhang, Y., Wei, S., Chen, Z., & Lin, G. (2021). Significant but short time assimilation of organic matter from decomposed exotic Spartina alterniflora leaf litter by mangrove polychaetes. Estuarine, Coastal and Shelf Science, 259, 107436.
47. Li, X., Yang, W., Sun, T., & Gaedke, U. (2021). Quantitative food web structure and ecosystem functions in a warm-temperate seagrass bed. Marine Biology, 168(5), 74.
48. Martinetto, P., Teichberg, M., & Valiela, I. (2006). Coupling of estuarine benthic and pelagic food webs to land-derived nitrogen sources in Waquoit Bay, Massachusetts, USA. Marine Ecology Progress Series, 307, 37-48.
49. Kinney, E. L., & Valiela, I. (2018). Spartina alterniflora δ15N as an indicator of estuarine nitrogen load and sources in Cape Cod estuaries. Marine pollution bulletin, 131, 205-211.
50. Logan, J. M. (2018). Salt marsh aboveground production in New England estuaries in relation to nitrogen loading and environmental factors. Wetlands, 38(6), 1327-1340.
51. Drake, D. C., Peterson, B. J., Deegan, L. A., Harris, L. A., Miller, E. E., & Warren, R. S. (2008). Plant nitrogen dynamics in fertilized and natural New England salt marshes: a paired 15N tracer study. Marine Ecology Progress Series, 354, 35-46.
52. Philben, M., & Benner, R. (2013). Reactivity of hydroxyproline-rich glycoproteins and their potential as biochemical tracers of plant-derived nitrogen. Organic geochemistry, 57, 11-22.
53. Tremblay, L., & Benner, R. (2006). Microbial contributions to N-immobilization and organic matter preservation in decaying plant detritus. Geochimica et Cosmochimica Acta, 70(1), 133-146.
54. Cole, M. L., Kroeger, K. D., McClelland, J. W., & Valiela, I. (2005). Macrophytes as indicators of land‐derived wastewater: Application of a δ15N method in aquatic systems. Water Resources Research, 41(1).
55. McClelland, J. W., & Valiela, I. (1998). Linking nitrogen in estuarine producers to land‐derived sources. Limnology and Oceanography, 43(4), 577-585.
56. Fry, B., Lutes, R., Northam, M., Parker, P. L., & Ogden, J. (1982). A 13C/12C comparison of food webs in Caribbean seagrass meadows and coral reefs. Aquatic Botany, 14, 389-398.
57. Wozniak, A. S., Roman, C. T., Wainright, S. C., McKinney, R. A., & James-Pirri, M. J. (2006). Monitoring food web changes in tide-restored salt marshes: a carbon stable isotope approach. Estuaries and Coasts, 29, 568-578.
